# SARS-CoV Pathogenesis Is Regulated by a STAT1 Dependent but a Type I, II and III Interferon Receptor Independent Mechanism

**DOI:** 10.1371/journal.ppat.1000849

**Published:** 2010-04-08

**Authors:** Matthew B. Frieman, Jun Chen, Thomas E. Morrison, Alan Whitmore, William Funkhouser, Jerrold M. Ward, Elaine W. Lamirande, Anjeanette Roberts, Mark Heise, Kanta Subbarao, Ralph S. Baric

**Affiliations:** 1 Department of Epidemiology, University of North Carolina at Chapel Hill, Chapel Hill, North Carolina, United States of America; 2 Laboratory of Infectious Diseases, NIAID, NIH, Bethesda, Maryland, United States of America; 3 Department of Microbiology and Immunology, University of North Carolina at Chapel Hill, Chapel Hill, North Carolina, United States of America; 4 Department of Anatomic Pathology and Surgical Pathology, University of North Carolina at Chapel Hill, Chapel Hill, North Carolina, United States of America; 5 Comparative Medicine Branch, NIAID, NIH, Bethesda, Maryland, United States of America; 6 Laboratory of Immunopathology, NIAID, NIH, Bethesda, Maryland, United States of America; University of Washington, United States of America

## Abstract

Severe acute respiratory syndrome coronavirus (SARS-CoV) infection often caused severe end stage lung disease and organizing phase diffuse alveolar damage, especially in the elderly. The virus-host interactions that governed development of these acute end stage lung diseases and death are unknown. To address this question, we evaluated the role of innate immune signaling in protection from human (Urbani) and a recombinant mouse adapted SARS-CoV, designated rMA15. In contrast to most models of viral pathogenesis, infection of type I, type II or type III interferon knockout mice (129 background) with either Urbani or MA15 viruses resulted in clinical disease outcomes, including transient weight loss, denuding bronchiolitis and alveolar inflammation and recovery, identical to that seen in infection of wildtype mice. This suggests that type I, II and III interferon signaling play minor roles in regulating SARS pathogenesis in mouse models. In contrast, infection of STAT1−/− mice resulted in severe disease, high virus titer, extensive pulmonary lesions and 100% mortality by day 9 and 30 post-infection with rMA15 or Urbani viruses, respectively. Non-lethal in BALB/c mice, Urbani SARS-CoV infection in STAT1−/− mice caused disseminated infection involving the liver, spleen and other tissues after day 9. These findings demonstrated that SARS-CoV pathogenesis is regulated by a STAT1 dependent but type I, II and III interferon receptor independent, mechanism. In contrast to a well documented role in innate immunity, we propose that STAT1 also protects mice via its role as an antagonist of unrestrained cell proliferation.

## Author Summary

The SARS coronavirus is a highly pathogenic respiratory virus that caused the first epidemic of the 21^st^ century. During the epidemic ∼10% of those infected died and the elderly were particularly vulnerable. Severe cases developed acute lung injury with pulmonary fibrosis and Acute Respiratory Distress Syndrome (ARDS). Little is known about the molecular mechanisms governing its virus pathogenesis and high lethality. Using a mouse model of infection with the epidemic strain of SARS-CoV (Urbani) as well as a recombinant mouse adapted strain of SARS-CoV (rMA15), we showed that a protein normally associated with the innate immune response, STAT1, plays an important role in the development of severe end stage lung injury. However, the lack of a normal innate immune type I, type II and type III interferon response did not enhance virus pathogenesis. Our work suggests that STAT1 may play a key role in development of acute lung injury and other chronic lung pathology, most likely by affecting cell proliferation and wound repair pathways.

## Introduction

SARS Coronavirus (SARS-CoV) is a highly pathogenic respiratory virus that emerged in China during the winter of 2002 and infected about 8,000 people globally and resulted in ∼800 deaths, with greatly increased mortality rates in persons over 50 years of age (WHO). On initial isolation of SARS-CoV from infected patients, it was identified as a novel Group 2 Coronavirus and the genetic mechanisms governing the increased pathogenicity of the virus remain undefined [1],[2]. In severe cases, SARS-CoV infection rapidly progressed to acute respiratory distress syndrome (ARDS) during the acute phase of infection or to an organizing phase diffuse alveolar damage following virus clearance; two clinically devastating end stage lung diseases. The molecular mechanisms governing these severe end stage lung disease outcomes are unknown, although similar pathologies have been reported following H5N1 and 1918 influenza virus infection.

The innate immune response is a key first line of defense against invading pathogens and is dependent on various signaling pathways and sensors that ultimately induce hundreds of anti-viral proteins to establish a suboptimal environment for replication and spread of invading pathogens [3],[4]. During virus infection the type I interferon (IFN) induction and signaling machinery is key to the initiation of this response. IFN induced from either infected cells or dendritic cells can activate an antiviral state in neighboring cells to signal that a viral infection is under way[5]. Not surprisingly, virus infections (mouse hepatitis virus, influenza virus, RSV, alphaviruses, flaviviruses, etc.) of rodents that lack type I or type II IFN regulatory networks result in increased pathogenesis and mortality, documenting the key role IFNs play in regulating disease outcomes[6]–[12].

Given the importance of the IFN system in regulating virus growth, many highly pathogenic viruses encode proteins that antagonize components of the innate immune system. The Ebola virus encodes VP35 which blocks STAT1 signaling[13],[14], influenza NS1 blocks IRF3 activation[15],[16] and V proteins from the Nipah and Hendra viruses induce STAT1 degradation[17]. Several IFN antagonist genes are encoded in the SARS-CoV genome and target IFN sensing and signaling and NFKB induction[18]–[21]. However, the role of IFN in regulating SARS-CoV pathogenesis *in vivo* is less clear. From microarray studies using RNA from PBMC's for 50 SARS patients, Cameron et. al. observed a robust type I IFN response early in the disease and predicted that it was essential for viral clearance and clinical recovery[22]. In addition to a strong IFN response, genes such as CCL2 (MCP-1) and CXCL10 (IP-10) that are typically induced by IFN were also up-regulated in patients at early times in SARS-CoV disease. Interestingly, Cameron and colleagues suggested that the IFN response that served to protect recovered SARS patients could become a dysregulated cytokine storm in severe cases and contribute to increased disease and a suboptimal adaptive immune response. The innate immune response to SARS-CoV also changes with age of the host; 12 month old BALB/c mice respond to SARS-CoV with an exacerbated and faster innate immune induction than 6 to 8 week old mice, potentially explaining their increased susceptibility and lung pathology following infection[23],[24].

Several studies have demonstrated the importance of the innate response in viral clearance. Respiratory syncytial virus (RSV) and SARS-CoV require MyD88 to produce an effective immune response to control severe infection in the lung [25],[26]. The Mx gene product plays an important role in the susceptibility of rodents to influenza virus infection and could regulate severity of SARS-CoV disease *in vivo* as well[27]. Glass *et al* demonstrated that SARS-CoV was cleared from the lungs of wildtype C57BL/6 mice and strains that lacked that T, B and NK cells with similar kinetics, suggesting that the innate immune response alone is sufficient for viral clearance [28]. Hogan et. al. also demonstrated that STAT1, a key modulator of IFN α/β, λ, γ signaling, was required for the resolution of wildtype SARS-CoV infection, once again indicating the importance of the innate response in the clearance of SARS-CoV [29].

In this paper, we compare the pathogenesis of both the epidemic virus (Urbani) and an isogenic, highly pathogenic mouse-adapted SARS-CoV (rMA15) in different mouse strains, each deficient in different innate immune signaling components. Specifically, we tested the role of type I, type II, and type III IFN and STAT1 in protection from SARS-CoV infection. We have found that absence of the IFN α/β, IFNγ and IFNλ, receptors alone or in some instances together, had a limited impact on pathogenicity and clearance of the non-lethal and lethal strains of SARS-CoV in mice. However, STAT1 deficient mice show increased susceptibility, prolonged virus shedding and mortality following infection with either virus. Importantly, STAT1−/− animals developed an organizing phase DAD, similar to lesions noted in severe late stage human cases of SARS. Our data reveals a new mechanistic pathway by which STAT1 regulates the severity of viral pathogenesis in the lung. We show that SARS-CoV pathogenesis is STAT1 dependent but independent of type I, II and III IFN signaling, and we provide evidence consistent with an essential role for STAT1 in the control of SARS-CoV replication, cell proliferation, wound repair and progression to severe organizing phase DAD and lethal disease.

## Results

### IFNα/β and IFNγ receptor knockout mice and 129 WT mice survive SARS-CoV infection

**Figure 1:**
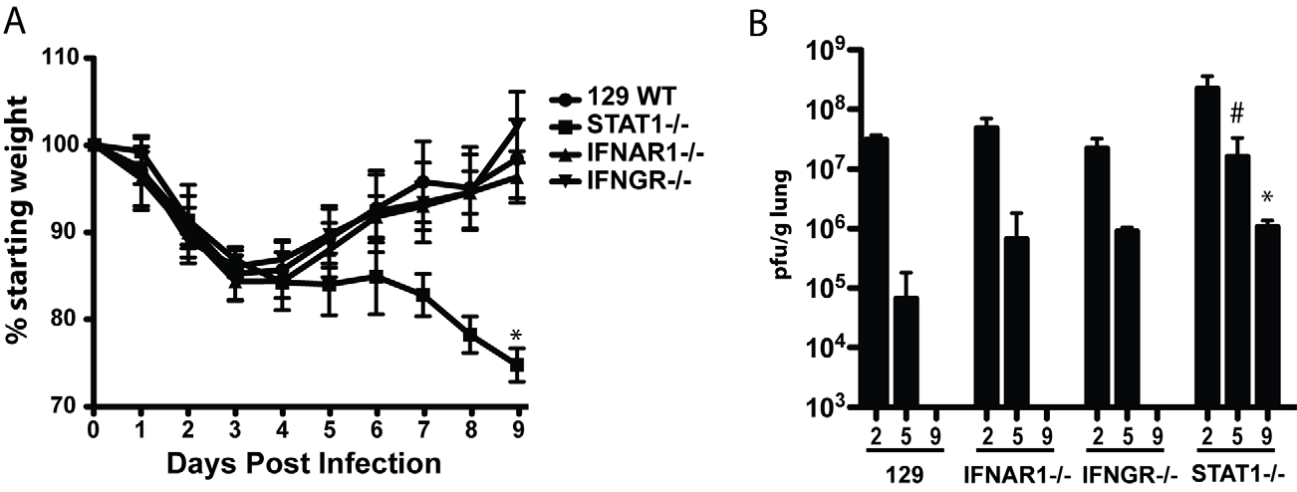
Mouse adapted SARS-CoV (rMA15) infection of 129 WT, IFNAR1−/−, IFNGR−/− and STAT1−/− mice. A. 10 week old female 129 WT, IFNAR1−/−, IFNGR−/− and STAT1−/− mice were infected intranasally with 1×10^5^ pfu/ml rMA15 virus. Individual mice were weighed daily and their average weight change from day 0 is presented. 10 mice per timepoint and strain were used in each group. The * =  a p value of .001. B. At days 2, 5 and 9 post-infection, groups of mice described in A were sacrificed and lungs were dissected, homogenized and supernatant used to titer virus in a plaque assay on Vero cells. Plotted is the average pfu/g of lung for 5 mice per group for each timepoint. The #  =  a p value of .005 and the *  =  a p value of .001. Each is a comparison of Day 9 WT compared to Day 9 STAT1−/−.

To understand the role of type I (IFNα/β) and type II IFN (IFNγ) in the response to SARS-CoV infection we infected IFN receptor −/− mice with either a late phase SARS-CoV (Urbani) virus or a recombinant isogenic mouse-adapted virus (rMA15) that contained six virulence modifying mutations [30], anticipating that the mouse-adapted virus may display more prominent pathogenicity than the Urbani virus. The Urbani virus and the equivalent recombinant icSARS viruses are not lethal in 10-wk old BALB/c, C57BL6 and 129 WT mice, typically replicating in the lungs with a peak titer at 2 days post-infection (dpi) before being cleared over the next 2–4 days without clinical disease or mortality [26],[30]. In contrast, the rMA15 virus is lethal in 10-wk old BALB/c mice causing greater than 20% weight loss by 4 days post-infection and death by 4–5 days post-infection. We infected 129 WT, Type I IFN receptor knockout mice (IFNAR1−/−) and Type I/II double IFN receptor knockout mice (IFNAGR−/−) with the Urbani virus (Figure S1) and 129 WT, IFNAR1−/− and IFNGR−/− with the rMA15 virus (Figure 1).

In 129 WT, IFNAR1−/− and IFNGR−/− mice, clinical findings including weight loss data and morbidity were identical after infection with rMA15. 129 WT, IFNAR1−/− and IFNGR−/− mice infected with rMA15 virus lost ∼15% of their weight by day 4 and then steadily recovered over the next 5 days (Figure 1). In contrast, Urbani virus infected mice continued to gain weight through the course of infection (Figure S1). In both cases, virus titers in the lungs peaked at day 2 post-infection, approaching titers of ∼5.0×10^7^ pfu or TCID_50_/g (rMA15 or Urbani, respectively), and all were rapidly cleared by day 9 post-infection. Although virus titers were ∼5-fold higher in IFNAR1−/− mice than titers in 129 WT mice on day 5 post-infection, we found that IFNAR1−/− and IFNGR−/− mice showed no increase in susceptibility, pathogenesis or histological outcomes to rMA15 or Urbani virus infection. To examine the importance of cooperative IFN pathways in disease, IFN receptor double knockout (IFNAGR−/−) mice were infected with the Urbani virus; no increase in virus titer or weight loss was seen compared to 129 WT mice or the single IFN receptor knockout mice (Figure S1). These data suggest that neither type I nor type II IFN receptors are critical for the regulation of SARS-CoV infection and pathogenic outcomes in mice.

### STAT1 protects mice from rMA15 and Urbani virus infection

In contrast to the results from IFN receptor knockout mice, previous studies have suggested that STAT1 −/− mice do not clear the Urbani virus by day 22 post-infection[29]. We infected STAT1−/− mice to evaluate whether the absence of a downstream IFN signaling protein results in increased susceptibility to SARS-CoV infection, and to determine the course of infection and pathologic changes associated with the virulent mouse-adapted virus. Importantly, STAT1−/− mice infected with rMA15 virus were more susceptible to disease than 129 WT, IFNAR1−/− or IFNGR−/− mice. Following rMA15 virus infection, STAT1−/− mice lost 15% of their starting weight by day 4 and continued to lose weight through day 9 post-infection (Figure 1). As weight loss approached 30%, the mice were moribund and succumbed to lethal infection. In stark contrast and consonant with earlier reports, STAT1−/− mice infected with Urbani virus initially gained weight as 129 WT mice did, through day 12 post-infection (Figure S1). However, over the next 15 days they displayed worsening clinical disease and lost significant body weight. They did not recover by day 29 post-infection, and 30% of them died.

Lung virus titers in STAT1−/− mice were also higher at each timepoint tested compared to the titers seen in IFN receptor knockout and 129 WT mice, (Figure 1B). When infected with rMA15 virus, STAT1−/− mice showed higher peak virus titers in the lungs at day 2 (∼10^8^ pfu/g) that remained as high as >10^6^ pfu/g at day 9 post-infection, while 129 WT mice had cleared the virus by 9 days. Urbani virus infected STAT1−/− mice also showed increased and sustained virus replication in the lungs as late as 15 days post-infection while virus was detectable only through day 5 post-infection in the 129 WT mice (Figure S1E). Taken together, these data suggest that a STAT1-dependent, IFN type I and II receptor-independent pathway plays a key role in regulating viral clearance and survival following SARS-CoV infection.

### Pulmonary disease associated with rMA15 virus infection in 129 WT and IFN receptor and STAT1 knockout mice

**Figure 2:**
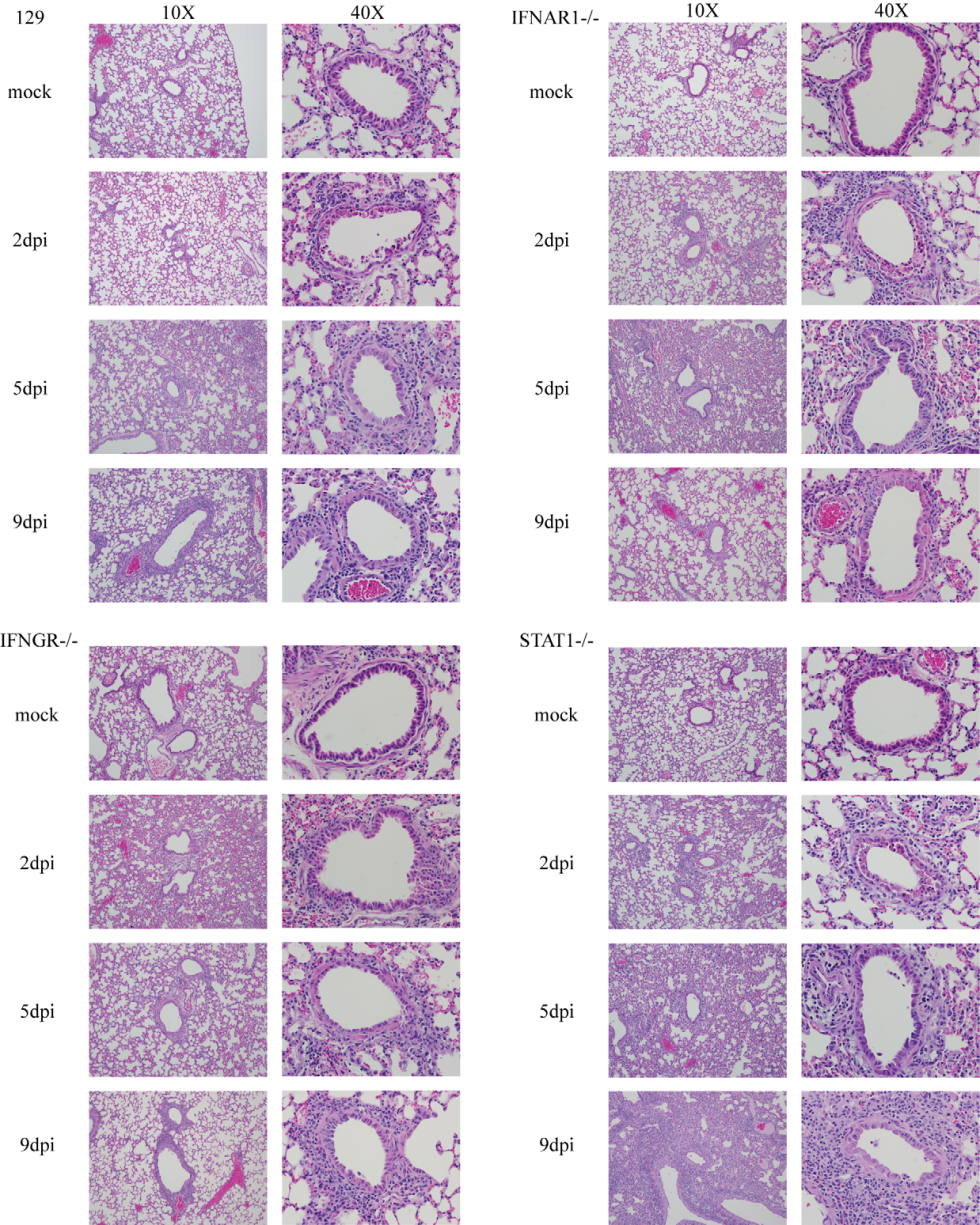
Histopathology of rMA15 virus infected mouse strains. Mice were sacrificed at days 2, 5 and 9 post-infection for histopathological analysis. Lung sections were stained with H&E and photomicrographs of individual airways are shown in the figure. The left side of each matched pair is a 10X picture and the right side is 40X.

**Figure 3:**
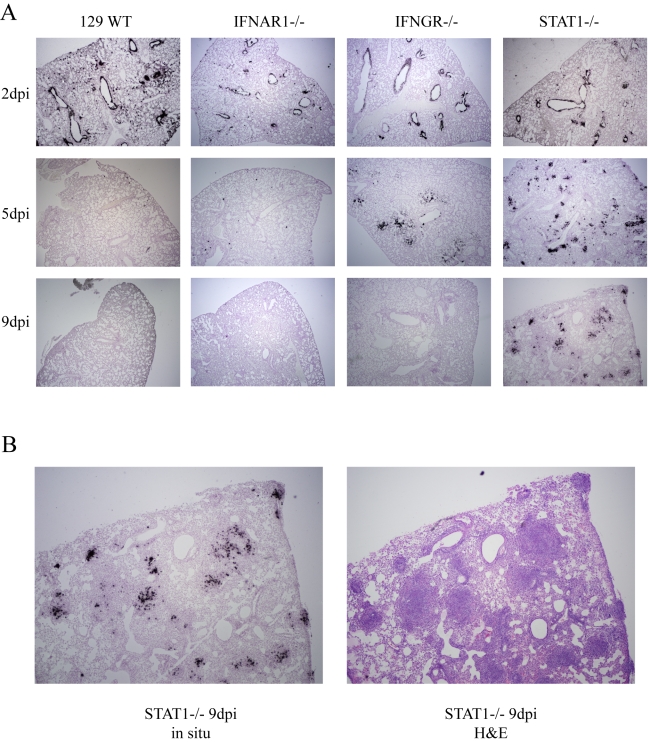
*In situ* hybridization of rMA15 infected mouse lungs. A. Paraffin embedded mouse lungs where sectioned and probed with S^35^ labeled probe complementary to SARS-CoV nucleocapsid RNA. Representative samples from days 2, 5 and 9 post-infection (dpi) are shown. Not shown are slides probed with a non-specific probe, which showed no labeling at all. Mock infected samples also showed no labeling. B. High magnification image of a 9 day post-infection STAT1−/− mouse lung with a corresponding H&E stained slide next to it. Note that areas with *in situ* signal overlap with areas of infiltration in the H&E stained section.

Lungs from the various mouse strains infected with SARS-CoV were analyzed for severity of histopathology (Figure 2). In the 129 WT mice, rMA15 virus caused a denuding bronchiolitis at 2 days post-infection with significant apoptosis of airway epithelial cells (noted by multilobed nuclei, condensed chromatin and nuclear blebbing), accumulation of apoptotic debris within the airways and perivascular cuffing caused predominately by lymphocytes. Similar lesions have been noted in BALB/c and C57BL6 mice[26]. rMA15 virus infection was primarily localized in airway epithelium at day 2 post-infection (Figure 3) and did not disseminate to other areas of the lung or respiratory tract. Some perivascular cuffing was noted in the vasculature by day 2 post-infection as well. By day 5, the denuding bronchiolitis, obstruction of the conducting airways by apoptotic debris and apoptosis of the airway epithelium were rarely observed, although perivascular cuffing and a mixed inflammatory response with lymphocytic infiltration of eosinophils, neutrophils and macrophages was more prominent. By day 9 post-infection, the remaining inflammation caused by rMA15 virus infection was primarily found in peribronchiolar areas (Figure 2).

Histologic changes in the lungs of IFNAR1−/− and IFNGR−/− mice were similar to 129 WT mice; the inflammation found in lung sections was temporally related to viral titer. At day 2 post-infection, a denuding bronchiolitis characterized by significant apoptosis and cell death in the airway epithelium was observed and conducting airways were obstructed by apoptotic debris. A mixed infiltrate composed of lymphocytes, neutrophils and eosinophils was seen surrounding the bronchial epithelium. By day 5 post-infection, significant inflammation was evident throughout the lungs and alveoli, resulting in a mild to moderate pneumonitis. As noted in lungs from 129 WT mice, the inflammation was clearing by day 9 post-infection; minimal inflammatory infiltrates remained in the periphery of the lungs and minor peribronchial lymphocytes remained.

At day 2 post-infection with rMA15 virus, lung lesions in STAT1−/− mice were indistinguishable from those seen in 129 WT, IFNAR1−/− and IFNGR−/− infected animals. By day 5 post-infection, lung lesions were more severe in STAT1−/− mice, consistent with persistently high virus titers (Figure 2). Two features were notable; first, the extent of inflammation was more severe in STAT1−/− mice, with a much greater number of macrophages infiltrating into all areas of the lung, a finding that was not seen in the other mouse strains. Second, thickening of the alveolar septa was seen throughout the lungs with perivascular and peribronchial thickening. By 9 days post-infection, the inflammation continued to increase in STAT1−/− mice while lung inflammation was subsiding in 129 WT, IFNAR1−/− and IFNGR−/− infected mice. In STAT1−/− mice, inflammation was pervasive throughout the lungs with densely packed infiltrating cells especially prominent throughout the periphery of the tissue. Macrophages continued to infiltrate with the majority residing in the alveolar interstitium. Interestingly, large foci containing densely packed fibroblasts, macrophages and lymphocytes were seen throughout the lungs and scattered atypical large cells were found throughout the foci. A focal mixed inflammatory infiltrate was found with extensive fibrin deposition. Pleuritis signified by a breakdown of the pleura was seen in most samples as well. This pathology was consistent with proliferative and organizing phase diffuse alveolar damage (DAD). It is noteworthy that similar pathologic lesions were seen in many fatal SARS cases [31]–[34].

### Pulmonary disease associated with SARS-CoV Urbani virus infection in mice

Lesions developing from days 2 to 9 in 129 WT, IFNAR1−/− and IFNAGR−/− mice inoculated with the Urbani virus were similar to those seen in rMA15-infected 129 WT mice. At days 2–3, there was diffuse bronchial and bronchiolar necrosis and migration of leukocytes (CD3+ T-cells, neutrophils and eosinophils) through vessel walls to peribronchiolar and perivascular areas but extension of lesions into the alveoli was not prominent (Figure S1F). Viral antigen was seen within the bronchial and bronchiolar epithelium (data not shown) as previously published[35]. By day 5, less necrosis was seen in the bronchiolar epithelium but peribronchiolar and perivascular cuffing remained. At day 9, necrosis was not observed and only minor perivascular cuffing was present. At days 15 and 22–29, the lungs were mostly normal except for minor perivascular and peribronchiolar cuffing of lymphocytes.

In STAT1−/− mice infected with the Urbani virus, there was a similar distribution of bronchial and bronchiolar lesions in the lung as seen in the 129 WT mice at day 3, but with more necrosis and inflammation. Edema was observed around peribronchiolar blood vessels and the larger inflammatory cell infiltrate contained many lymphocytes, and neutrophils with a few eosinophils and macrophages. Abundant viral antigen was seen in bronchiolar epithelium (data not shown). By day 5, the bronchiolar epithelium had foci of regeneration with little necrosis and the peribronchiolar and perivascular inflammation was less severe than at day 3. Plugs of cellular debris and fibrin filled some bronchioles. A blue tinge was noted in the perivascular edema fluid, suggesting early collagen deposition. Viral antigen was much less common in the epithelium but was abundant in the cellular debris in the airways. At day 9, inflammatory cells increased around residual inflammatory lesions, with abundant neutrophils and more macrophages in the lesions. Marked epithelial hyperplasia was also seen in these foci. Bronchiolitis obliterans was seen in a few airways and interstitial lesions developed around some airway lesions, one of which extended to the pleura and producing focal pleuritis. Much of the lung parenchyma was, however, histologically normal. Only a few cells or cell debris was seen expressing viral antigen. By day 15, a fibrinous pleuritis with pyogranulomatous lesions developed in 2 of 3 mice, with focal resolving parenchymal lesions including a few foci of chronic interstitial pneumonia but most of the lung parenchyma remained fairly normal. From day 15–24, a fibrinous peritonitis (Figure S2A), pleuritis and pyogranulomatous lesions in spleen, liver and omentum developed as the major lesions that likely contributed to illness and death in the STAT1−/− mice (Figure S2B-F). These lesions were characterized by a central area of necrosis with numerous neutrophils and an outer zone of macrophages. Viral antigen was found in some of the macrophages in these pyogranulomatous lesions (Figure S2D, E). Plasma cells became abundant in the splenic lesions by day 24 (Figure S2G). Abundant fibrosis (detected by Masson's trichrome stain) was seen in the splenic and liver lesions at day 24 (Figure S2F). The lungs of mice from days 15–24 were mostly normal with areas of residual chronic inflammation and a few pyogranulomas, some of which contained viral antigen. The pleura had nodules of pyogranulomatous and fibrinous inflammation.

### In situ hybridization of rMA15 virus infected lungs

We determined the localization of virus in infected lungs during the course of infection by *in situ* hybridization. We used a riboprobe complementary to the SARS-CoV nucleocapsid RNA that was labeled with radioactive nucleotides to visualize viral RNA in the tissue. *In situ* hybridization was performed on lungs from 129 WT, IFNAR1−/−, IFNGR−/− and STAT1−/− mice harvested at days 2, 5, and 9 post-infection. In all 4 strains of mice, the probe signal was predominantly localized in the airway epithelial cells at day 2 post-infection (Figure 3A). This correlated with the pathologic lesions in airway epithelial cells at day 2 post-infection. By day 5 post-infection, viral RNA was virtually eliminated from the control 129, and IFNAR1−/− mice and to a slightly lesser extent in IFNGR−/− mice. Occasionally, a few cells with viral RNA were noted in the periphery, consistent with the low titers of virus in these animals at day 5. By day 9, there was no viral RNA signal found in the lungs of 129 WT, IFNAR1−/− and IFNGR−/− mice, corresponding to the lack of viral replication at this time point. In contrast, lungs of STAT1−/− mice showed significant viral RNA signal throughout the lungs, including prominent distribution into the periphery of the lung at early and late times post-infection. Surprisingly, but consistent with the findings on histopathology, viral RNA signal was lost from the airway epithelial cells in the bronchioles by day 9 in STAT1−/− mice and was found throughout the periphery of the lungs, prominently focused in large focal compactions of cells. These foci correspond to the prominent focal lesions noted in H&E stained sections of the lung, which are predominantly composed of fibroblasts, macrophages and lymphocytes (Figure 3B). Interestingly, cellular debris can be seen in these foci that may represent lysed airway epithelial cells.

### Characterization of inflammatory cell infiltrates

We observed marked differences in pathology of lungs from 129 WT mice compared to STAT1−/− mice after infection with the rMA15 virus. To investigate whether STAT1 affected the inflammatory infiltrate, we isolated leukocytes from enzymatically disrupted mouse lungs harvested on day 8 post-infection and used cell surface markers to quantify macrophage (CD11c^−^/CD11b^+^/GR-1^int^/MHCII^+^), neutrophil (F480^−^/CD11b^+^/CD11c^−^/GR-1^+^), and eosinophil (CD11c^−^/Siglec^+^/GR-1^−^) populations in 129 WT and STAT1−/− mice. A greater than 10-fold difference in leukocyte numbers was found between 129 WT and STAT1−/− infected mice (Table 1). As shown in Table 1, the eosinophil population increased from ∼2% in 129 WT mice to ∼30% in STAT1−/− mice and neutrophils increased from 3% in 129 WT mice to 35% in STAT1−/− mice. Additionally, the number of macrophages in STAT1−/− mice was more than double that detected in 129 WT mice. These numbers are concordant with the histological findings by H&E staining at 9 days post-infection.

**Table 1:** Quantitation of the inflammatory cell infiltrate in the lungs of rMA15 virus infected STAT1−/− mice at 8dpi.

	Percent (number) of cells in the lungs of indicated strains of mice following mock or rMA15 infection			
	129 + PBS	STAT1−/− + PBS	129 + rMA15	STAT1−/− + rMA15
Total leukocytes isolated	12,702	14,823	13,211	160,561
Dendritic Cells	1.8% (236)	2.3% (110)	12.97% (1,713)	16.8% (26,902)
Macrophages	1.2% (154)	1.1% (53)	8.6% (1,137)	13.4% (21,490)
Neutrophils	2.6% (377)	3.3% (159)	2.2% (293)	30.8% (49,411)
Eosinophils	1.2% (150)	0.7% (337)	0.8% (107)	16.3% (26,119)

Analysis of inflammatory cell infiltrates during rMA15 infection of STAT1−/− mice. Lymphocytes from lungs of 129 WT and STAT1−/− mice infected with rMA15 or PBS for 8 days, were isolated and labeled with antibodies to identify inflammatory cell populations by flow cytometry. Macrophages were identified as CD11c^−^/CD11b^+^/GR-1^int^/MHCII^+^ cells, neutrophils as F480^−^/CD11b^+^/CD11c^−^/GR-1^+^ cells and eosinophils as CD11c^−^/Siglec^+^/GR-1^−^ cells. The percent of total starting lymphocytes is shown in the table for each cell type.

### Cytokine and chemokine protein and gene expression studies

**Figure 4:**
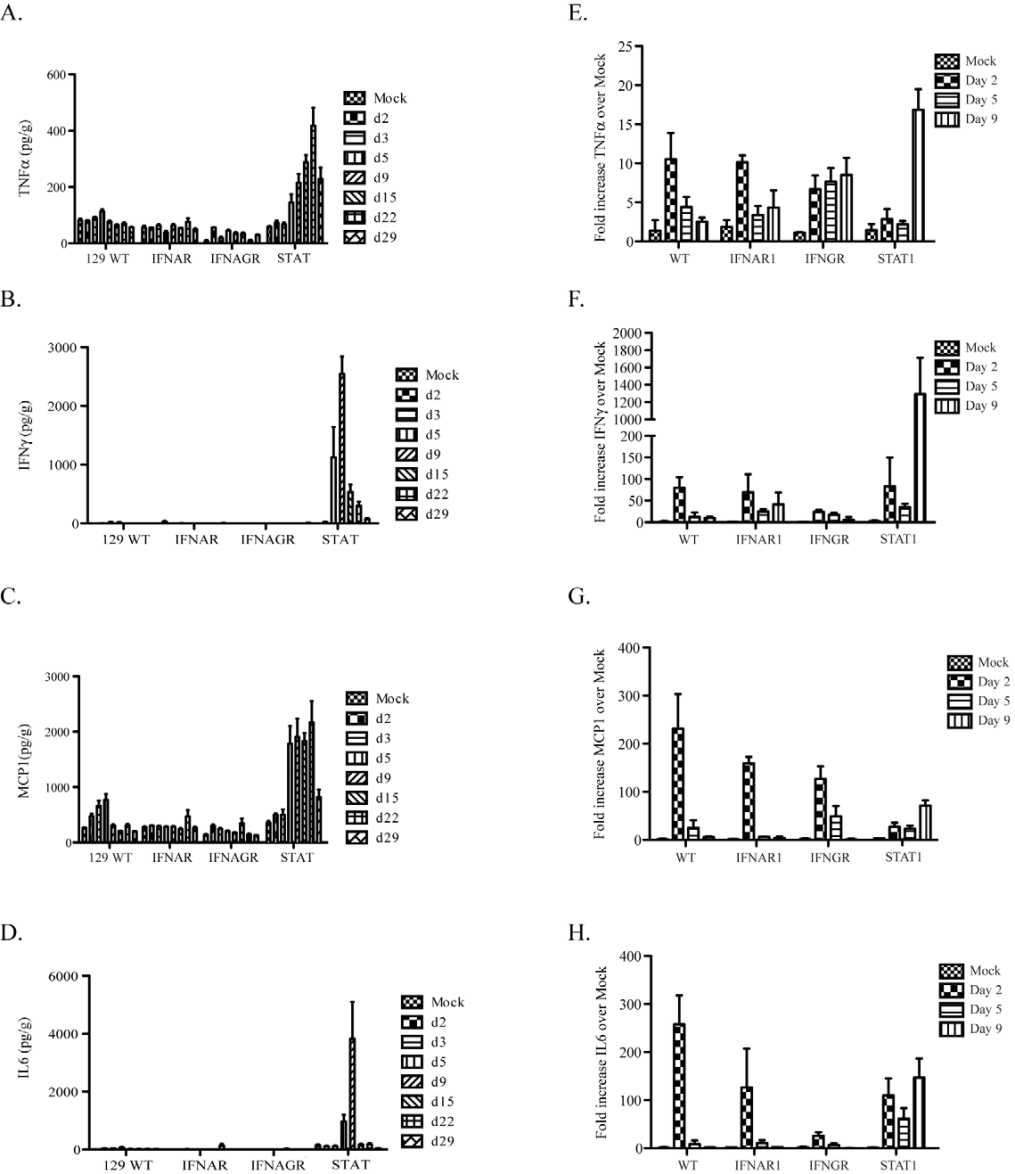
Protein and gene expression analysis of cytokines. A–D. A cytometric bead array was used to quantify the protein levels of cytokines in lung homogenates after infection with the Urbani virus. Lung samples were homogenized and protein levels of (A) TNFα, (B) IFNγ, (C) MCP1 and (D) IL6 were quantified. E–F. Real-time PCR analysis was performed on RNA from infected mouse lungs. Mice were harvested at days 2, 5 and 9 post-infection and lung RNA isolated by Trizol. cDNA was made from 1ug of RNA of each and used in real-time analysis and the results for 3 mice per group were combined. Fold increase over mock-infected lungs of each cytokine is graphed for (E) TNFα, (F) IFNγ, (G) MCP1 and (H) IL6.

We used real time RT-PCR and CBA analysis to quantify changes in mRNA and protein expression levels of several innate immune factors involved in pulmonary inflammation. Specifically, we compared the induction of several pro-inflammatory cytokines (TNFα, IL6, IFNγ and MCP1) for changes in expression during infection by analyzing lung homogenates by CBA (Figure 4A, B, C and D) or quantitative RT-PCR to measure levels of mRNAs in Urbani virus or rMA15 virus infected mice, respectively (Figure 4E, F, G and H).

In Urbani virus infected lungs, minimal changes were seen in 129 WT, IFNAR1−/−, IFNAGR−/− mice for all 4 cytokines (Figure 4A, B, C and D). However, in STAT1 −/− mice, significant increases in protein expression patterns were detected across different time points, peaking between 9 and 15 days post-infection. TNFα protein levels increased steadily from day 2 through day 29 post-infection while IL6 and IFNγ protein levels peaked at day 9, before reducing to levels seen in mock infected animals. MCP1 protein levels were increased between days 5 through 22 but diminished by day 29 post-infection.

Cytokine induction following rMA15 virus infection was compared with Urbani virus infection (Figure 4A–H). Comparing cytokine transcript levels on days 2, 5 and 9 post-infection, we noted some similarities and important differences. Following rMA15 virus infection, 129 WT mice showed a 10-fold induction of TNFα transcripts by day 2 post-infection, followed by progressively reduced levels of expression that returned to baseline by day 9. While IFNAR1−/− mice showed similar kinetics as 129 WT mice, infection in IFNGR−/− mice resulted in a continued rise in TNFα expression till day 9. In contrast, STAT1−/− mice had low baseline levels of TNFα expression through day 5, after which high levels of expression were noted at day 9, corresponding to the peak time of inflammation in the lungs, just prior to death.

IFNγ and MCP1 gene expression levels were similar in rMA15 virus-infected 129 WT, IFNAR1−/− and IFNGR-/mice. Transcript levels were induced by day 2 and then decreased through day 9. While STAT1−/− infected mice showed a similar trend of increased transcript levels at day 2, expression levels of IFNγ and MCP1 continued to increase through day 9 post-infection. Finally, IL6 expression showed similar kinetics in rMA15 virus infected 129 WT control and IFNAR1−/− mice with peaks at day 2 that decreased to baseline levels by day 5. IFNGR−/− mice showed similar kinetics, but with lower expression levels at day 2. Comparatively, induction of IL6 in STAT1−/− mice followed a different pattern, with high levels of expression at day 2 post-infection and persistent high level expression through day 9. IFNβ, IFNα4, IL28B, IL18, IRF1 and OAS1 were analyzed by Real-time PCR from lung RNA isolated during the rMA15 timecourse in WT, IFNAR−/− and STAT1−/− mice (Figure S3). IFNβ, IFNα4, IL28B, IRF1 and OAS1 show a peek of induction at 2dpi for all 3 strains with a reduction throughout the timecouse with a few exceptions. IFNβ, IFNα4 and OAS1 show either a sustained or late burst in expression of each in only the STAT1−/− mice. Interestingly, IL18 shows minimal induction (∼1.5 fold) in WT mice but even less in the IFNAR−/− and STAT1−/− mice. Thus, significant differences in cytokine expression patterns were noted between STAT1, WT and IFNAR deficient animals, where typically WT and IFNAR−/− mice had the same expression patters while STAT1−/− mice were typified by perceived loss of regulatory control and persistent high level expression.

### Type III IFN does not play a role in SARS-CoV pathogenesis

**Figure 5:**
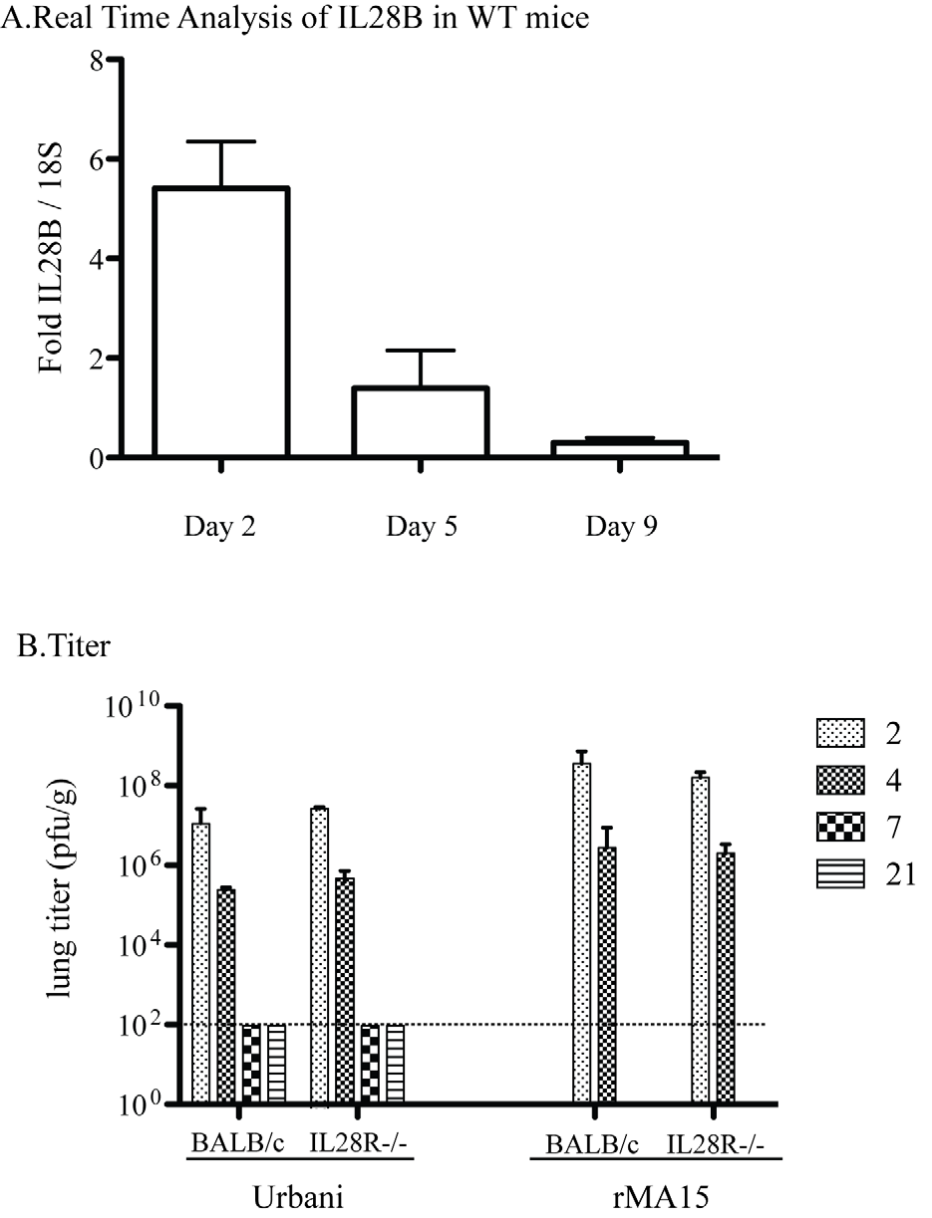
Pathogenesis of Urbani and rMA15 viruses in IL28Ra−/− mice. BALB/c and IL28Ra−/− mice were infected with 1×10^5^ pfu of either Urbani or rMA15 virus. A. Real time analysis of IL28B transcripts was performed on WT 129 infected mice at 2, 5 and 9 days post infection. Graphed is the fold increase over 18S RNA for each mouse with 5 mice at each timepoint averaged together. B. Titer of Urbani and rMA15 virus at each timepoint. Lungs were homogenized and supernatant of each lung was titered by plaque assay on Vero cells. Shown is the average titer from 5 mice at each timepoint. The level of detection is shown by a dotted line at 100 pfu/ml.

STAT1 is a mediator of Type III IFN (IFN L, Lambda) signaling in addition to Type I and II IFN signaling. IFNL is minimally but significantly upregulated during infection in WT 129 mice at day 2 post infection which decreases through the course of 9 days post infection (Figure 5A). The receptor for IFNL is a heterodimer of IL10Rb and IL28Ra. IL28Ra−/− mice have been generated on the BALB/c mouse background but not on the 129 WT mouse background. Thus, although direct comparison with the other mouse strains is not possible, experiments with SARS-CoV in these mice are still informative. As in the C57B6 background, the Urbani virus does not cause disease or weight loss but the virus replicates in the lungs of BALB/c mice. Virus reaches peak titer by day 2 post-infection and infection is resolved by day 7[36]. In contrast, rMA15 virus infection of BALB/c mice causes death by day 4 or 5 post-infection[30]. We hypothesized that if IFNL was important for protection from SARS-CoV infection, the virus would be more virulent in mice lacking the IFNL receptor and IFNL receptor knockout mice would show evidence of disease while normal BALB/c mice would not. Further, rMA15 virus infection of IL28Ra−/− mice may result in enhanced pathology with more weight loss, higher virus titer, or increased lung pathology.

This hypothesis was tested by inoculating control BALB/c mice and IL28Ra−/− mice with the Urbani virus, rMA15 virus or PBS. There was no weight loss in either BALB/c or IL28Ra−/− mice infected with the Urbani virus (data not shown), thus the lack of IFNλ signaling did not potentiate clinical disease. Lungs from Urbani virus infected BALB/c and IL28Ra−/− mice were harvested at 2, 4, 7 and 21 days post-infection. We found no difference between the titers of virus in the lungs of BALB/c and IL28Ra−/− mice (Figure 5B). Lungs from infected mice were analyzed by H&E staining. Urbani virus infection of IL28Ra−/− mice produced mild inflammation that peaked at day 2 and resolved by day 7 post-infection; replicating the pattern seen in BALB/c mice (Figure S4A).

rMA15 virus infection caused >20% weight loss and/or death by day 4-5 post-infection in BALB/c and similar findings in IL28Ra−/− mice (data not shown); both lost ∼20% weight by day 4 post-infection. Lungs were harvested at days 2 and 4 post-infection and no differences in virus titers were observed (Figure 5B). Both mouse strains showed peak virus titers at day 2 with a 2-3 log decrease by day 4. Lung pathology was analyzed by H&E staining. Both BALB/c and IL28Ra−/− mice showed similar high levels of inflammation at 2 and 4 days post-infection, but remarkably similar pathologic outcomes (Figure S4B). Taken together, we found no role for IFNλ in protection from infection with either the Urbani or rMA15 strains of SARS-CoV.

### IFNLR antibody in IFNAR1−/− mice

**Figure 6:**
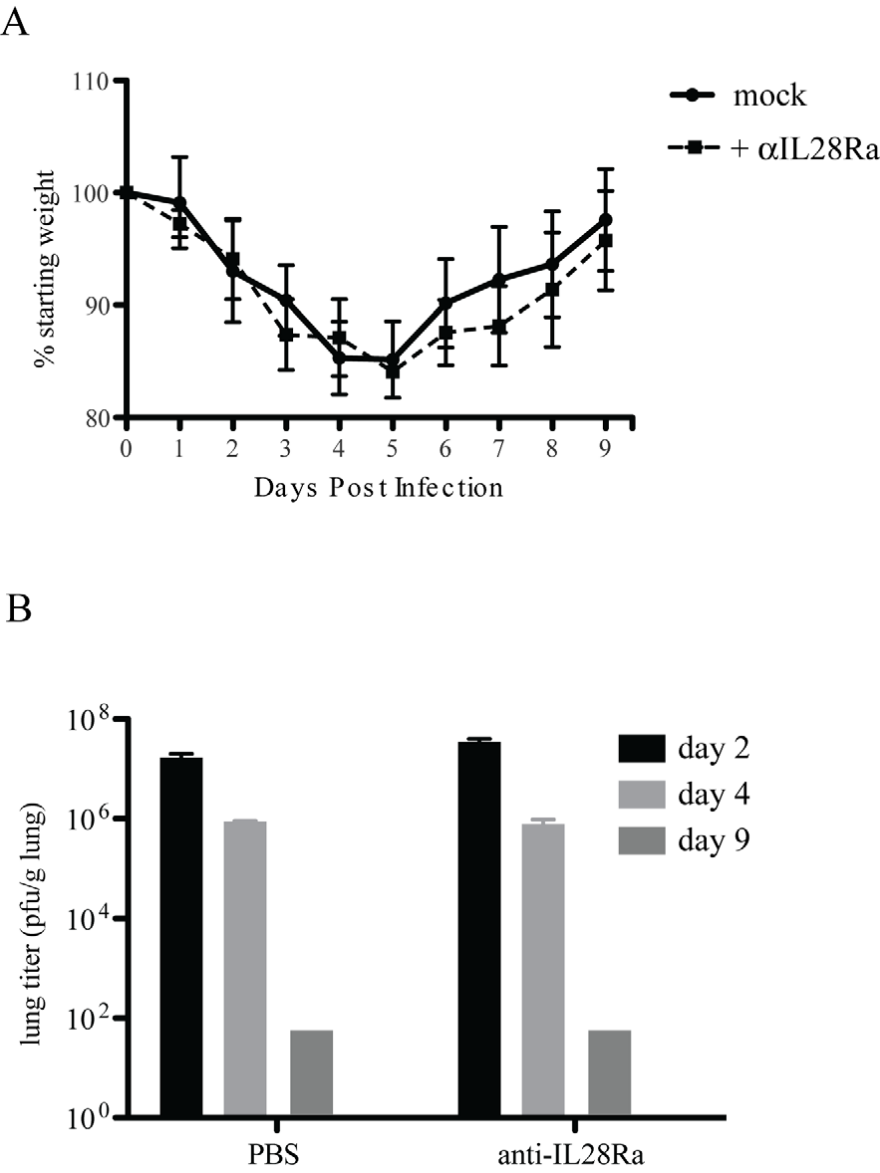
IFNL receptor antibody treatment of IFNAR1−/− mice. IFNAR1−/− mice were treated with 100µg of anti-IL28Ra antibody and infected with rMA15 virus. A. Mock infected (PBS) and antibody injected IFNAR1−/− mice were infected with 1×10^5^ pfu/ml rMA15 virus at day 0. Average weights for each group are plotted compared to starting weights (5 mice per group). B. Mouse lungs were harvested at days 2, 4 and 9 post-infection. Virus titer in lung homogenates were determined on Vero cells. 5 mice were used per timepoint and the average titer of the group of 5 mice is shown.

BALB/c and 129 WT mice respond differently to rMA15 virus infection; rMA15 is lethal in BALB/c mice but only causes transient weight loss in 129/Sv mice. To analyze the effects of knocking out both Types I and III IFN signaling pathways, we treated IFNAR1−/− mice (on the 129 background) with neutralizing antibodies to IL28Ra, the receptor used by IFNλ. Mice were injected with 100 µg of anti-IL28Ra antibody at days -1, 1, 3, 5 and 7 days post-infection as described in the literature[37]. We found no difference in weight loss or pathogenesis of rMA15 in these mice compared to mice injected with PBS (Figure 6). Mice showed 15% weight loss by day 4 post-infection but recovered by day 9, ending at their starting weight. Lungs were analyzed at days 2, 4 and 9 post-infection and showed no difference in pathology compared to IFNAR1−/− mice (data not shown). Both groups of mice showed epithelial cell denudation at 2 day post-infection and repair and clearance by day 9. Virus titers were only slightly increased over PBS injected mice but the difference was not statistically significant. This suggests that the inhibition of both Type I and Type III signaling in mice does not increase the pathogenesis of the rMA15 virus and neither protect 129 mice from disease.

## Discussion

### Paradigm of IFN signaling and protection

SARS-CoV infection in humans rapidly progressed from an atypical pneumonia, to acute phase diffuse alveolar damage and ARDS during the first 10 days of acute lung injury. In many patients this is followed by the development of an organizing phase DAD after virus clearance. Both pathologies were associated with severe clinical outcomes and death, especially prominent in the elderly. The molecular mechanisms and virus-host signaling networks that regulate these progressive end stage lung diseases are unknown but are of considerable importance, given the global disease burden associated with them. Previous studies in our laboratory have demonstrated that aged mice infected with recombinant viruses encoding S glycoproteins from early phase or zoonotic SARS-CoV strains developed ARDS, characterized by hyaline membranes and DAD. In this report, we show that STAT1 deficient mice are especially prone to the development of organizing phase DAD.

The innate immune system plays a central role in regulating early host responses to virus infection and promoting adaptive immune responses. The Type I, II and III IFNs are typically produced by different cell populations and use distinct membrane bound receptors to gain entry into cells; Type I uses IFNAR1, Type II uses IFNGR and Type III uses IL28Ra/IL10Rb. However, all three share a common cytoplasmic signaling protein, called STAT1, that is translocated to the nucleus and induces expression of multiple, overlapping IFN regulated genes (ISGs)[38]. Deletion of any or all of the signaling components involved in the STAT1 signaling pathway diminishes the innate immune response to pathogens and increases susceptibility to several bacterial and viral agents. Mice lacking IFN receptors show increased susceptibility to West Nile[9], influenza[12],[39], Ebola[11], Friend virus[40], RSV[7],[8] and Poliovirus[41] (reviewed in [42]). Moreover, the same phenotypes are noted in STAT1 −/− mice, demonstrating a key role of the entire innate immune pathway in regulating disease severity, viral titers, and pathology[10]. In stark contrast, we demonstrate the paradoxical finding that SARS-CoV Urbani and rMA15 viruses induce severe end stage lung disease by a STAT1 dependent mechanism that is independent of IFN receptor type I, II and III signaling. The data point to a novel mechanism by which STAT1 function regulates disease severity in the lung following SARS-CoV induced acute lung injury.

### New paradigms for STAT1 regulation of acute lung injury

In contrast to the existing paradigm, SARS-CoV infection is successfully controlled and cleared in IFNAR1−/−, IFNGR−/−, IFNAGR−/− and IFNLR deficient mice while deletion of STAT1 leads to increased virus replication, morbidity and mortality following infection with either the human epidemic strain of SARS-CoV (Urbani) or a mouse adapted strain (rMA15). These results suggest a novel mechanism of STAT1 regulation of severe end stage lung disease following SARS-CoV infection that is independent of it's roles in IFN signaling and ISG expression. Although it is possible that cooperative combinations of several IFNs (IFNα/β, IFNγ, IFNλ) that signal through STAT1 are required to regulate SARS-CoV infection, we feel that the possibility exists that STAT1's role in controlling cell proliferation and wound healing may be the base cause of the increased disease seen in STAT1−/− mice. Nevertheless, the development of triple knockouts affecting all 3 IFN receptors would address this mechanistic possibility.

### Alternative roles for STAT1

STAT1 functions a key gatekeeper in mediating IFNα/β, IFNγ and IFNλ signaling into the nucleus to induce overlapping but distinct ISGs. Less well appreciated in viral pathogenesis studies, STAT1 also plays key roles in cell cycle arrest and cell proliferation[43]–[45]. Thus, STAT1 defects may augment viral lung disease by several potential mechanisms. STAT1 was shown to be an important controller of tumor formation in several types of cancers including lung, colon, pancreatic and brain cancers[46]–[48] and its role in cell proliferation has been studied in the context of pulmonary fibrosis[43]. STAT1−/− mouse fibroblasts showed increased proliferation when exposed to growth factors compared to WT mouse fibroblasts. Additionally, STAT1−/− mice demonstrated a greater susceptibility to chemically induced pulmonary fibrosis via bleomycin treatment. These data suggested that outside of the innate immune response, STAT1 may function as a key regulator of cell proliferation and of the wound healing response[49]. Our data also suggest that STAT1 may regulate the wound healing response following acute lung injury associated with viral infection, similar to its cell cycle regulation seen in other models of disease.

### STAT1 and SARS-CoV

Lungs of STAT1−/− mice infected with rMA15 virus progressed to an early stage pulmonary fibrosis-like disease in 9 days. The acute lung injury (ALI) seen in these lungs approximates the damage seen in ARDS that was often seen in severe cases of SARS in humans[50], especially in the elderly. We found perivascular cuffing, collapse of alveolar parenchyma, invasion and amplification of macrophages, neutrophils, eosinophils and fibroblasts and most importantly, extensive fibrin deposition throughout the lungs. These pathological findings mirror those seen in ALI, pulmonary fibrosis and ARDS.

SARS-CoV, like many highly pathogenic viruses, expresses several proteins that antagonize the IFN sensing and amplification network. SARS-CoV ORF6 blocks STAT1 nuclear import[18], PLP blocks IRF3 activation[51], NSP7[51], NSP15[51], ORF3b and N have been shown to be IFN antagonists as well[19]. Importantly, these antagonists only function in the context of SARS-CoV infection within discrete permissive cells. This suggests that in infected cells, the multiple pathways that inhibit IFN signaling may create essentially a STAT−/− environment which may contribute to the further pathology seen during disease. As has been described with influenza, it is likely that a cytokine storm significantly contributes to increased pathogenic outcomes by targeting non-infected cells. The loss of STAT1 in other cells of the knockout mouse may contribute as we have described here, to loss of wound healing control and induction of fibrosis, leading to the development of the lethal disease state after SARS-CoV infection.

The molecular mechanisms governing SARS-CoV pathogenesis have only recently begun to be evaluated using mice with targeted genetic defects challenged with the rMA15 virus. Sheahan et. al. identified a role for Myd88, an adapter protein of TLR signaling, and RAG1, necessary for antibody production, in protection from infection and disease. Myd88 −/− mice showed an enhanced susceptibility to rMA15 virus infection in C57/B6 mice[26] and RAG1−/− mice showed no increased morbidity and mortality from rMA15 virus infection, viral replication in the lungs was detectable through 28 days post-infection[26]. These data suggest that an intricate balance between the innate and adaptive immune response is necessary for protection from SARS-CoV infection. We are currently working on understanding how these two processes interact in the host. Using proteomic and microarray analyses Cameron *et al* [22] showed that individuals who survived SARS-CoV infection had controlled innate immune, ISG and cytokine responses while individuals who progressed to severe disease demonstrated an uncontrolled innate immune response, with high levels of ISG and immunoglobulin expression, increased cytokine responses and poor antibody responses to the spike protein. The cause of this lack of regulation is not understood.

### Comparison of Urbani to rMA15 virus

Infection with the epidemic (Urbani) and mouse adapted strains of SARS-CoV caused increased pathology in the STAT1−/− mice. Thus, it seems likely that the mouse adapted mutations in rMA15 enhanced the intrinsic pathogenic properties of SARS-CoV to produce severe end stage lung disease in the mice. However, SARS-CoV Urbani spreads from the respiratory tract into the spleen and liver of STAT1−/− mice. This suggests that although the damage may have been induced by viral infection, the pathology results from the host's response to the infection, though it is not clear whether this represents a normal pattern of spread or whether mutations have evolved in the virus that promote spread and distribution to other organs. Similar findings were seen in autopsy samples from individuals who died from SARS. Airways were intact and regenerated while acute cases showed spread to the outer pulmonary parenchyma[52].

The similarities between STAT1−/− mice infected with SARS-CoV and elderly individuals infected with SARS-CoV during the epidemic are intriguing. Recent work has shown that in cells from aged hosts, STAT1 signaling cascades are less responsive to stimuli; STAT1 signaling in aged macrophages were hypo-responsive to IFNγ[49]. As observed in our study, IFNγ expression increases substantially in STAT1−/− mice. We hypothesize that this may be in response to a lack of negative feedback via the STAT1 signaling pathway. During the SARS epidemic, aged individuals were the cohort with most severe disease and highest mortality rates[53]. We propose that altered STAT1 signaling in aged individuals may have lead to increased susceptibility to severe disease.

We cannot rule out the possibility that all 3 types of IFN receptors are redundant for SARS-CoV mediated disease and that only by deleting all 3 types of receptors will be observe an increase in pathogenesis. Proof must await the availability of a mouse strain containing the deletion of all three receptors. However we find that the type of pathology produced in STAT1−/− mice by both WT and rMA15 SARS-CoV demonstrates a role for STAT1 in non-innate immune processes that resemble those produced in severely infected SARS-CoV patients.

We propose a new potential pathway by which STAT1 regulates end stage lung disease following viral infection. We hypothesize that the increased susceptibility of STAT1−/− mice results from different roles of STAT1 in the cell. First, loss of STAT1 results in a deficient IFN response that results in higher titers of virus in the lungs. Secondly, loss of STAT1 allows for unregulated cell proliferation in response to the innate immune response, causing enhanced damage to the lungs and death of the animals. Our findings also point to an increasing role of the cell damage response to viral infection that can be potential targets for therapy in highly pathogenic respiratory infections.

## Methods

### Ethics statement

All mice in this study were treated following IACUC guidelines. For infection mice were pre-treated with Ketamine and Xylazine as an anesthetic. Mice were euthanized if their weight dropped below 20% of their starting weight or if clinical symptoms warranted it per our IACUC approvals. Animal housing and care and experimental protocols were in accordance with all UNC-Chapel Hill Institutional Animal Care and Use Committee guidelines or NIH guidelines, depending where the experiments were performed.

### Viruses and cells

Vero E6 cells were grown in MEM (Invitrogen, Carlsbad, CA) supplemented with 10% FCII (Hyclone, South Logan, UT) and gentamicin/kanamycin (Gibco-BRL). Stocks of the biological SARS-CoV (Urbani), recombinant SARS-CoV (icSARS) and mouse-adapted SARS-CoV (rMA15) were propagated and titered on Vero or Vero E6 cells and cryopreserved at −80°C until use as described [30]. All experiments with infectious virus were performed in a Class II biological safety cabinet in a certified biosafety level 3 laboratory containing redundant exhaust fans with personnel wearing protective equipment including Tyvek suits, hoods, and HEPA-filtered powered air-purifying respirators (PAPRs) as described [30].

### Mice

129S6/SvEv wildtype and STAT1−/− mice (catalog number 002045-M-F) were obtained from Taconic Farms (Germantown, NY). For the mouse adapted SARS-CoV infections, Type I IFN receptor deficient (IFN alpha/beta receptor) (IFNAR1−/−) mice were bred in at the UNC mouse facility (Chapel Hill, North Carolina). Type II IFN receptor deficient (IFN gamma receptor) (IFNGR−/−) mice (stock number 002702) were purchased from The Jackson Laboratories (Bar Harbor, ME). For the Urbani virus infections, IFNAR1−/− mice were obtained as a gift from Dr. Joan Durbin at Ohio State University and IFN alpha/beta/gamma receptor double knockout (IFNAGR−/−) mice were bred at the NIH animal facility (Bethesda, MD). IFN-lambda receptor knockout mice (IL28Ra−/−, Zymogen, Seattle, Washington) were bred at the UNC Chapel Hill animal facility. Animal housing and care and experimental protocols were in accordance with all UNC-Chapel Hill Institutional Animal Care and Use Committee guidelines or NIH guidelines, depending where the experiments were performed. All animal studies were conducted in Animal Biosafety Level 3 laboratories using SealSafe Hepa-filtered caging and personnel wore personal protective equipment, including Tyvek suits and hoods and positive pressure HEPA-filtered air respirators. 10 week old mice were anesthetized with a mixture of ketamine/xylazine or isoflurane and intranasally infected with either PBS alone or 10^5^ pfu/50 µl rMA15 or the recombinant or biological epidemic virus, icSARS or Urbani, in PBS (Invitrogen, Carlsbad, CA). Mice were monitored at 24 h intervals for virus-induced morbidity and mortality. Subsets of mice were euthanized at days 2, 5, and 9 post-infection (dpi) for characterization of rMA15 infection, while the less pathogenic Urbani virus infected animals were sampled on days 2, 3, 5, 9, 15, 22 and 29 post-infection. All tissues were analyzed for histopathology changes and for viral titers.

### Viral replication in organs

To quantify the amount of infectious virus in tissues from rMA15 infection, each organ was weighed, placed in 0.5 ml DPBS, homogenized, and titered via plaque assay on Vero E6 cells as previously described [26]. For Urbani infection, supernatants of 10% (w/v) lung homogenates were prepared and titrated on Vero cell monolayers in 24- and 96-well plates as previously described [40]. Virus titers are expressed as TCID_50_ per g of tissue. The lower limit of detection was 10^1.5^ TCID_50_/g.

### Histological analysis

Lung tissues were fixed in PBS/4% para-formaldehyde, pH 7.3, embedded in paraffin, and 5 µm sections were prepared by the UNC histopathology core facility. To determine the extent of inflammation, sections were stained with hematoxylin and eosin (H & E) and scored in a blinded manner.

### BD Cytometric Bead Array (CBA) for protein expression

Supernatants of 20% (weight/volume) lung homogenates were used for detection of cytokines and chemokines using BD CBA kits (BD Biosciences) according to the manufacturer's instructions. The lower limit of detection for each protein is included in the kit protocol.

### In situ hybridization

^35^S-UTP-labeled riboprobes specific to the N gene of SARS-CoV (Urbani) or to the EBER2 gene from Epstein-Barr virus (negative control probe) were generated with an SP6-specific MAXIscript *in vitro* transcription kit (Ambion) and *in situ* hybridization was performed as described previously[26]. Briefly, deparaffinized tissue sections were hybridized with 5×10^4^ cpm/µl of ^35^S-labeled riboprobes overnight. Tissues were washed, dehydrated through graded ethanol, coated in NTB autoradiography emulsion (Kodak), and incubated at −80°C for 7 days. Following development, sections were counterstained with hematoxylin and silver grain deposition was analyzed by light microscopy. rMA15-specific signal was determined by comparing silver grain deposition on parallel sections hybridized with the ^35^S-labeled riboprobe complementary for the EBER2 gene of Epstein-Barr virus.

### Real-time PCR analysis

Lungs from mock or SARS-CoV infected mice were removed and homogenized directly in 1 ml of Trizol reagent (Invitrogen) and total RNA was isolated following manufacturer's instructions. Complementary cDNA was generated from 1 µg of total RNA using 250 ng random primers (Invitrogen) and Superscript II reverse transcriptase (Invitrogen). Real-time PCR experiments were performed using Taqman gene expression assays and an ABI Prism 7300 (Applied Biosystems). 18S rRNA was used as an endogenous control to use for normalization in all assays. The relative fold induction of amplified mRNA were detected using the Ct method. Taqman primer sets used were 18S (#Hs03003631 Applied Biosystems), IFNγ (Mm00801778 Applied Biosystems), TNFa (Mm99999068 Applied Biosystems), MCP1 (Mm99999056 Applied Biosystems), IFNB (Mm00439552 Applied Biosystems), IFNA4 (Mm00833969 Applied Biosystems), IL28B (Mm00663660 Applied Biosystems), IL18 (Mm00434225 Applied Biosystems), IRF1 (Mm01288574 Applied Biosystems) and OAS1 (Mm00449297).

### Flow cytometry

Mice were inoculated as described above, sacrificed by exsanguination at 2 and 4 days post-infection, and lungs were perfused via cardiac puncture with 1× PBS. Lungs were dissected, minced, and incubated for 2 hrs with vigorous shaking at 37°C in digestion buffer [RPMI, 10% FBS, 15 mM HEPES, 2.5 mg/ml collagenase A (Roche), 1.7 mg/ml DNase I (Sigma)]. Cells were passed through a 40 micron cell strainer, resuspended in RPMI media, layered on 5 ml lympholyte-M (Cedarlane), and centrifuged 30 min at 2500 rpm. Cells were collected, washed in wash buffer (1× HBSS, 15 mM HEPES), and total viable cells were determined by trypan blue exclusion. Isolated cells were incubated with anti-mouse FcγRII/III (2.4G2; BD Pharmingen) for 20 min on ice and then stained in FACS staining buffer (1× HBSS, 1% FBS, 2% normal rabbit serum) with the following antibodies from eBioscience: anti-F4/80-FITC, anti-Gr-1-PE, anti-CD11b-APC, anti-CD11c-PE, anti-Ly-6C-FITC, anti-CD3-FITC, anti-CD8-APC, anti-CD4-PerCP, and anti-NK1.1-PE. Cells were fixed overnight in 2% paraformaldehyde and analyzed on a Cyan cytometer (Dako) using Summit software.

### Statistical analyses

Percent starting weights, viral titers and inflammatory cell numbers were evaluated for statistically significant differences by the non-parametric Mann-Whitney test within GraphPad Prism or unpaired t-tests using GraphPad InStat3 software. P values of ≤0.05 were considered significant.

## Supporting Information

Figure S1 Urbani virus infection of 129 WT (A), IFNAR1−/− (B), IFNAGR−/− (C) and STAT1−/− (D) mice. Mice were infected with the Urbani virus and weighed each day for 29 days. Shown is their average weight change from day 0 across each group (n>5). E. Mice were harvested at each timepoint and lung homogenates determined in Vero cells are expressed as mean TCID_50_ per gram of lung for each group. F. Urbani virus infected mouse lungs stained with H&E. A representative lung from each mouse strain across the timecourse is shown. Note the increased inflammation in the lungs of STAT1−/− mice through the course of disease, especially at day 15 post-infection.
(6.33 MB TIF)

Figure S2 Gross pathology of lungs and peripheral organs of Urbani virus infected mice. A. Gross lesions in organs from an Urbani virus infected STAT1−/− mouse 15 dpi showing a fibrinous exudate on the spleen and nodules on the liver. B. Lung from a STAT1−/− mouse 24 dpi displaying a pyogranulomatous nodule. H&E, X100. C. Spleen from a STAT1−/− mouse 24 dpi showing the central area of the pyogranuloma with neutrophils on left and macrophages on the right side. H&E, X400. D. Liver of a STAT1−/− mouse stained with anti-SARS-CoV antibody showing viral antigen in macrophages on edges of pyogranuloma. Hematoxylin, IHC, X400. E. Spleen of STAT1−/− mouse 24 dpi, showing many pyogranulomas. H&E, X40. F. Spleen of STAT1−/− mouse at 24 dpi stained with Mason's Trichome stain, showing collagen (blue) in zones around the pyogranulomas, X100. G Spleen of STAT1−/− mouse infected with Urbani virus 22 dpi stained with anti-kappa light chain, showing numerous plasma cells adjacent to the pyogranuloma. Hematoxylin, IHC, X200.
(7.14 MB TIF)

Figure S3 Real Time analysis on innate immune response genes. RNA from rMA15 infected WT, IFNAR−/− and STAT1−/− mice was extracted and used for Realtime analysis of (A) IFNB, (B) IFNA, (C) IL18B, (D) IL18, (E) and (F) OAS1. Graphed is the fold increase over 18S rRNA for each sample at 2, 5 and 9 days post infection. For each timepoint, RNA from 5 mice was harvested and the average is shown with the standard deviation of each sample set.
(0.48 MB EPS)

Figure S4 A. Lungs from mice infected with Urbani virus were harvested at days 2, 4, 7 and 21 post-infection (n = 5 for each timepoint). Shown are representative sections from IL28Ra−/− mouse lungs stained with H&E at each timepoint. B. Mice were infected with rMA15 virus and lungs were harvested at days 2 and 4 post-infection. The infection was lethal for both BALB/c and IL28Ra−/− mice by day 4 post-infection. Uninfected BALB/c mouse lungs are shown as a comparison.
(4.65 MB TIF)

## References

[1] Donnelly CA, Ghani AC, Leung GM, Hedley AJ, Fraser C (2003). Epidemiological determinants of spread of causal agent of severe acute respiratory syndrome in Hong Kong.. Lancet.

[2] Drosten C, Gunther S, Preiser W, van der Werf S, Brodt HR (2003). Identification of a novel coronavirus in patients with severe acute respiratory syndrome.. N Engl J Med.

[3] Levy DE, Garcia-Sastre A (2001). The virus battles: IFN induction of the antiviral state and mechanisms of viral evasion.. Cytokine Growth Factor Rev.

[4] Seth RB, Sun L, Chen ZJ (2006). Antiviral innate immunity pathways.. Cell Res.

[5] Takaoka A, Yanai H (2006). Interferon signalling network in innate defence.. Cell Microbiol.

[6] Ryman KD, White LJ, Johnston RE, Klimstra WB (2002). Effects of PKR/RNase L-dependent and alternative antiviral pathways on alphavirus replication and pathogenesis.. Viral Immunol.

[7] Durbin JE, Johnson TR, Durbin RK, Mertz SE, Morotti RA (2002). The role of IFN in respiratory syncytial virus pathogenesis.. J Immunol.

[8] Johnson TR, Mertz SE, Gitiban N, Hammond S, Legallo R (2005). Role for innate IFNs in determining respiratory syncytial virus immunopathology.. J Immunol.

[9] Keller BC, Fredericksen BL, Samuel MA, Mock RE, Mason PW (2006). Resistance to alpha/beta interferon is a determinant of West Nile virus replication fitness and virulence.. J Virol.

[10] Durbin JE, Hackenmiller R, Simon MC, Levy DE (1996). Targeted disruption of the mouse Stat1 gene results in compromised innate immunity to viral disease.. Cell.

[11] Bray M (2001). The role of the Type I interferon response in the resistance of mice to filovirus infection.. J Gen Virol.

[12] Durbin JE, Fernandez-Sesma A, Lee CK, Rao TD, Frey AB (2000). Type I IFN modulates innate and specific antiviral immunity.. J Immunol.

[13] Basler CF, Wang X, Muhlberger E, Volchkov V, Paragas J (2000). The Ebola virus VP35 protein functions as a type I IFN antagonist.. Proc Natl Acad Sci U S A.

[14] Reid SP, Leung LW, Hartman AL, Martinez O, Shaw ML (2006). Ebola virus VP24 binds karyopherin alpha1 and blocks STAT1 nuclear accumulation.. J Virol.

[15] Salvatore M, Basler CF, Parisien JP, Horvath CM, Bourmakina S (2002). Effects of influenza A virus NS1 protein on protein expression: the NS1 protein enhances translation and is not required for shutoff of host protein synthesis.. J Virol.

[16] Yuan W, Krug RM (2001). Influenza B virus NS1 protein inhibits conjugation of the interferon (IFN)-induced ubiquitin-like ISG15 protein.. Embo J.

[17] Rodriguez JJ, Parisien JP, Horvath CM (2002). Nipah virus V protein evades alpha and gamma interferons by preventing STAT1 and STAT2 activation and nuclear accumulation.. J Virol.

[18] Frieman M, Yount B, Heise M, Kopecky-Bromberg SA, Palese P (2007). Severe acute respiratory syndrome coronavirus ORF6 antagonizes STAT1 function by sequestering nuclear import factors on the rough endoplasmic reticulum/Golgi membrane.. J Virol.

[19] Kopecky-Bromberg SA, Martinez-Sobrido L, Frieman M, Baric RA, Palese P (2007). Severe acute respiratory syndrome coronavirus open reading frame (ORF) 3b, ORF 6, and nucleocapsid proteins function as interferon antagonists.. J Virol.

[20] Wathelet MG, Orr M, Frieman MB, Baric RS (2007). Severe acute respiratory syndrome coronavirus evades antiviral signaling: role of nsp1 and rational design of an attenuated strain.. J Virol.

[21] Zhao J, Falcon A, Zhou H, Netland J, Enjuanes L (2009). Severe acute respiratory syndrome coronavirus protein 6 is required for optimal replication.. J Virol.

[22] Cameron MJ, Ran L, Xu L, Danesh A, Bermejo-Martin JF (2007). Interferon-mediated immunopathological events are associated with atypical innate and adaptive immune responses in patients with severe acute respiratory syndrome.. J Virol.

[23] Rockx B, Baas T, Zornetzer GA, Haagmans B, Sheahan T (2009). Early Upregulation of ARDS Associated Cytokines Promote Lethal Disease in an Aged Mouse Model of SARS-CoV Infection.. J Virol.

[24] Baas T, Roberts A, Teal TH, Vogel L, Chen J (2008). Genomic analysis reveals age-dependent innate immune responses to severe acute respiratory syndrome coronavirus.. J Virol.

[25] Haynes LM, Moore DD, Kurt-Jones EA, Finberg RW, Anderson LJ (2001). Involvement of toll-like receptor 4 in innate immunity to respiratory syncytial virus.. J Virol.

[26] Sheahan T, Morrison TE, Funkhouser W, Uematsu S, Akira S (2008). MyD88 is required for protection from lethal infection with a mouse-adapted SARS-CoV.. PLoS Pathog.

[27] Haller O, Arnheiter H, Gresser I, Lindenmann J (1981). Virus-specific interferon action. Protection of newborn Mx carriers against lethal infection with influenza virus.. J Exp Med.

[28] Glass WG, Subbarao K, Murphy B, Murphy PM (2004). Mechanisms of host defense following severe acute respiratory syndrome-coronavirus (SARS-CoV) pulmonary infection of mice.. J Immunol.

[29] Hogan RJ, Gao G, Rowe T, Bell P, Flieder D (2004). Resolution of primary severe acute respiratory syndrome-associated coronavirus infection requires Stat1.. J Virol.

[30] Roberts A, Deming D, Paddock CD, Cheng A, Yount B (2007). A Mouse-Adapted SARS-Coronavirus Causes Disease and Mortality in BALB/c Mice.. PLoS Pathog.

[31] Lo AW, Tang NL, To KF (2006). How the SARS coronavirus causes disease: host or organism?. J Pathol.

[32] Hwang DM, Chamberlain DW, Poutanen SM, Low DE, Asa SL (2005). Pulmonary pathology of severe acute respiratory syndrome in Toronto.. Mod Pathol.

[33] Paul NS, Roberts H, Butany J, Chung T, Gold W (2004). Radiologic pattern of disease in patients with severe acute respiratory syndrome: the Toronto experience.. Radiographics.

[34] Ketai L, Paul NS, Wong KT (2006). Radiology of severe acute respiratory syndrome (SARS): the emerging pathologic-radiologic correlates of an emerging disease.. J Thorac Imaging.

[35] Roberts A, Paddock C, Vogel L, Butler E, Zaki S (2005). Aged BALB/c mice as a model for increased severity of severe acute respiratory syndrome in elderly humans.. J Virol.

[36] Subbarao K, McAuliffe J, Vogel L, Fahle G, Fischer S (2004). Prior infection and passive transfer of neutralizing antibody prevent replication of severe acute respiratory syndrome coronavirus in the respiratory tract of mice.. J Virol.

[37] Kotenko SV, Gallagher G, Baurin VV, Lewis-Antes A, Shen M (2003). IFN-lambdas mediate antiviral protection through a distinct class II cytokine receptor complex.. Nat Immunol.

[38] Schindler C, Darnell JE (1995). Transcriptional responses to polypeptide ligands: the JAK-STAT pathway.. Annu Rev Biochem.

[39] Koerner I, Kochs G, Kalinke U, Weiss S, Staeheli P (2007). Protective role of beta interferon in host defense against influenza A virus.. J Virol.

[40] Gerlach N, Schimmer S, Weiss S, Kalinke U, Dittmer U (2006). Effects of type I interferons on Friend retrovirus infection.. J Virol.

[41] Kuss SK, Etheredge CA, Pfeiffer JK (2008). Multiple host barriers restrict poliovirus trafficking in mice.. PLoS Pathog.

[42] Katze MG, He Y, Gale M (2002). Viruses and interferon: a fight for supremacy.. Nat Rev Immunol.

[43] Walters DM, Antao-Menezes A, Ingram JL, Rice AB, Nyska A (2005). Susceptibility of signal transducer and activator of transcription-1-deficient mice to pulmonary fibrogenesis.. Am J Pathol.

[44] Antao-Menezes A, Turpin EA, Bost PC, Ryman-Rasmussen JP, Bonner JC (2008). STAT-1 signaling in human lung fibroblasts is induced by vanadium pentoxide through an IFN-beta autocrine loop.. J Immunol.

[45] Bromberg J, Darnell JE (2000). The role of STATs in transcriptional control and their impact on cellular function.. Oncogene.

[46] Frank DA (1999). STAT signaling in the pathogenesis and treatment of cancer.. Mol Med.

[47] Klampfer L (2006). Signal transducers and activators of transcription (STATs): Novel targets of chemopreventive and chemotherapeutic drugs.. Curr Cancer Drug Targets.

[48] Levitzki A, Mishani E (2006). Tyrphostins and other tyrosine kinase inhibitors.. Annu Rev Biochem.

[49] Yoon P, Keylock KT, Hartman ME, Freund GG, Woods JA (2004). Macrophage hypo-responsiveness to interferon-gamma in aged mice is associated with impaired signaling through Jak-STAT.. Mech Ageing Dev.

[50] Lau YL, Peiris JS (2005). Pathogenesis of severe acute respiratory syndrome.. Curr Opin Immunol.

[51] Frieman M, Ratia K, Johnston RE, Mesecar AD, Baric RS (2009). Severe acute respiratory syndrome coronavirus papain-like protease ubiquitin-like domain and catalytic domain regulate antagonism of IRF3 and NF-kappaB signaling.. J Virol.

[52] Nicholls JM, Butany J, Poon LL, Chan KH, Beh SL (2006). Time course and cellular localization of SARS-CoV nucleoprotein and RNA in lungs from fatal cases of SARS.. PLoS Med.

[53] Chan-Yeung M, Xu RH (2003). SARS: epidemiology.. Respirology.

