# Supplementary figures and images for: SARS-CoV Pathogenesis Is Regulated by a STAT1 Dependent but a Type I, II and III Interferon Receptor Independent Mechanism
Source: PLoS Pathog. 2010 Apr 8;6(4):e1000849. doi: 10.1371/journal.ppat.1000849 (PMC2851658; doi:10.1371/journal.ppat.1000849)

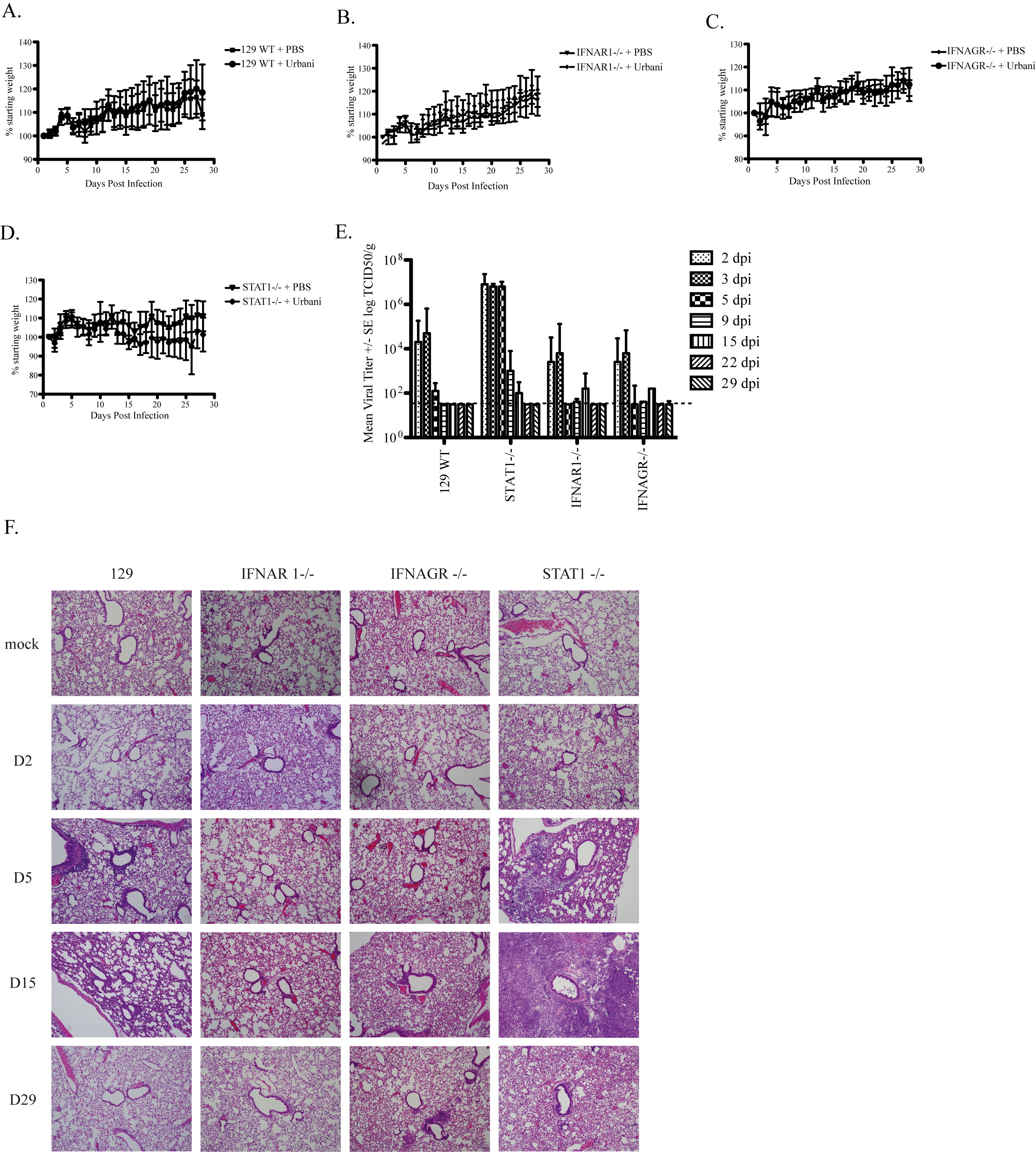

Supplement: Figure S1 — Urbani virus infection of 129 WT (A), IFNAR1−/− (B), IFNAGR−/− (C) and STAT1−/− (D) mice. Mice were infected with the Urbani virus and weighed each day for 29 days. Shown is their average weight change from day 0 across each group (n>5). E. Mice were harvested at each timepoint and lung homogenates determined in Vero cells are expressed as mean TCID50 per gram of lung for each group. F. Urbani virus infected mouse lungs stained with H&E. A representative lung from each mouse strain across the timecourse is shown. Note the increased inflammation in the lungs of STAT1−/− mice through the course of disease, especially at day 15 post-infection. (6.33 MB TIF) [file ppat.1000849.s001.tif]

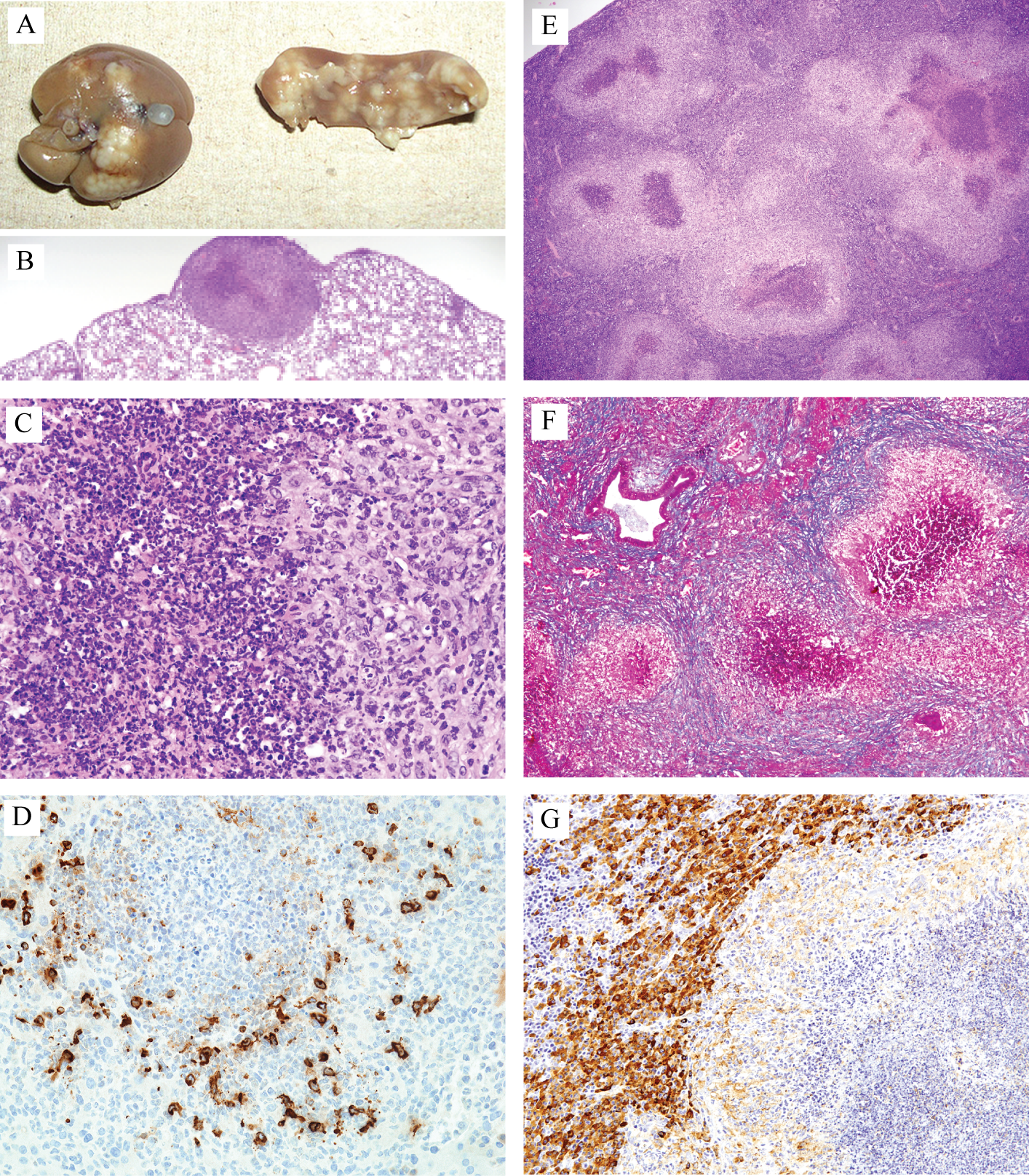

Supplement: Figure S2 — Gross pathology of lungs and peripheral organs of Urbani virus infected mice. A. Gross lesions in organs from an Urbani virus infected STAT1−/− mouse 15 dpi showing a fibrinous exudate on the spleen and nodules on the liver. B. Lung from a STAT1−/− mouse 24 dpi displaying a pyogranulomatous nodule. H&E, X100. C. Spleen from a STAT1−/− mouse 24 dpi showing the central area of the pyogranuloma with neutrophils on left and macrophages on the right side. H&E, X400. D. Liver of a STAT1−/− mouse stained with anti-SARS-CoV antibody showing viral antigen in macrophages on edges of pyogranuloma. Hematoxylin, IHC, X400. E. Spleen of STAT1−/− mouse 24 dpi, showing many pyogranulomas. H&E, X40. F. Spleen of STAT1−/− mouse at 24 dpi stained with Mason's Trichome stain, showing collagen (blue) in zones around the pyogranulomas, X100. G Spleen of STAT1−/− mouse infected with Urbani virus 22 dpi stained with anti-kappa light chain, showing numerous plasma cells adjacent to the pyogranuloma. Hematoxylin, IHC, X200. (7.14 MB TIF) [file ppat.1000849.s002.tif]

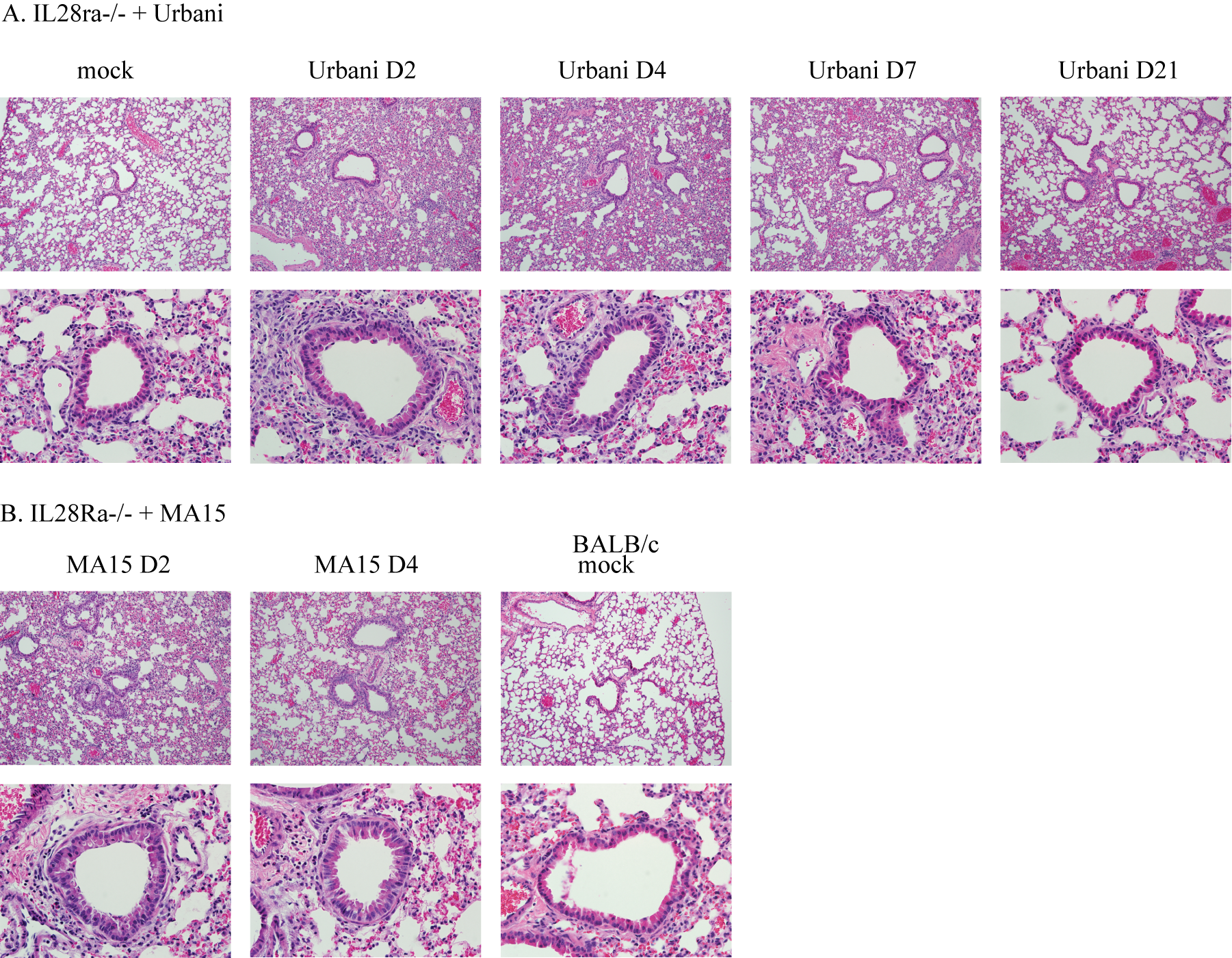

Supplement: Figure S4 — A. Lungs from mice infected with Urbani virus were harvested at days 2, 4, 7 and 21 post-infection (n = 5 for each timepoint). Shown are representative sections from IL28Ra−/− mouse lungs stained with H&E at each timepoint. B. Mice were infected with rMA15 virus and lungs were harvested at days 2 and 4 post-infection. The infection was lethal for both BALB/c and IL28Ra−/− mice by day 4 post-infection. Uninfected BALB/c mouse lungs are shown as a comparison. (4.65 MB TIF) [file ppat.1000849.s004.tif]
